# Of Mice and Men: Divergence of Gene Expression Patterns in Kidney

**DOI:** 10.1371/journal.pone.0046876

**Published:** 2012-10-03

**Authors:** Lydie Cheval, Fabien Pierrat, Rabary Rajerison, David Piquemal, Alain Doucet

**Affiliations:** 1 Unité Mixte de Recherche 872, Université Paris 6 and Institut National de la Santé et de la Recherche Médicale, Paris, France; 2 Equipe de Recherche 7226, Centre National de la Recherche Scientifique, Paris, France; 3 Skuld-Tech, Montpellier, France; Ecole Normale Supérieure de Lyon, France

## Abstract

Since the development of methods for homologous gene recombination, mouse models have played a central role in research in renal pathophysiology. However, many published and unpublished results show that mice with genetic changes mimicking human pathogenic mutations do not display the human phenotype. These functional differences may stem from differences in gene expression between mouse and human kidneys. However, large scale comparison of gene expression networks revealed conservation of gene expression among a large panel of human and mouse tissues including kidneys. Because renal functions result from the spatial integration of elementary processes originating in the glomerulus and the successive segments constituting the nephron, we hypothesized that differences in gene expression profiles along the human and mouse nephron might account for different behaviors. Analysis of SAGE libraries generated from the glomerulus and seven anatomically defined nephron segments from human and mouse kidneys allowed us to identify 4644 pairs of gene orthologs expressed in either one or both species. Quantitative analysis shows that many transcripts are present at different levels in the two species. It also shows poor conservation of gene expression profiles, with less than 10% of the 4644 gene orthologs displaying a higher conservation of expression profiles than the neutral expectation (p<0.05). Accordingly, hierarchical clustering reveals a higher degree of conservation of gene expression patterns between functionally unrelated kidney structures within a given species than between cognate structures from the two species. Similar findings were obtained for sub-groups of genes with either kidney-specific or housekeeping functions. Conservation of gene expression at the scale of the whole organ and divergence at the level of its constituting sub-structures likely account for the fact that although kidneys assume the same global function in the two species, many mouse “models” of human pathologies do not display the expected phenotype.

## Introduction

Research in biology largely relies on the availability of efficient model organisms. Their choice is dictated by the conservation of mechanisms between species and by general traits that include their amenability to growing/breeding (small size, short life-cycle, non-specialized living requirements) and to genetic manipulation (inbred strains, methods for transformation/recombination and gene extinction). Besides these general properties, some models are chosen owing to specific advantages: the genome compactness of *Saccharomyces cerevisiae* and *Arabidopsis thaliana* and their low proportion of junk DNA make their genetic study easier; the ease of generating mutations and identifying their morphological consequences are the two main properties that made the common fruit fly *(Drosophila melanogaster)* the most studied organism, particularly in genetics and developmental biology; the small and fixed number of constituting cells and the large fraction of those undergoing apoptosis during the life cycle has made the nematode *Caenorhabditis elegans* a widely used model for studying development and apoptosis; the transparency of the zebrafish (*Danio rerio)* body during early development facilitates analysis of its internal anatomy.

Choice of mammalian models is dictated by anthropomorphic considerations: they must resemble *Homo sapiens* as much as possible in order to help decipher the development, physiology and pathophysiology of our species. For obvious reasons, primates like the Rhesus macaque (*Macaca mulatta*) have often been chosen for cognition studies. In other fields of physiology, the rat *(Rattus norvegicus)* has long been a universal model owing to the larger size of its organs relative to those of the mouse. Nevertheless, in the last decades, the development of efficient methods for homologous gene recombination in mice *(Mus musculus)* [1] and lower breeding costs have boosted the use of mice, which have supplanted rats in most fields of physiology and pathophysiology. However, these strong scientific and economic considerations have somehow overshadowed the first requirement of a mammalian model, i.e. the similarity of its physiological properties to those of humans.

In the fields of renal physiology and pathophysiology, considerable advances were made possible by the development of gene invalidation (knock out) and expression of wild type or mutated genes (knock in) by homologous gene recombination in mice. However, there is a wealth of differences in kidney function and pathology between men and mice. For example a) the level of basal proteinuria observed in mice is viewed as pathological in humans, b) mice are resistant to many drugs that induce glomerulopathies in humans, c) alterations in mouse renal function hardly increase their blood pressure by more than 15 mm Hg. There are also many examples where knock-out mice for a gene whose loss-of-function mutations are responsible for a disease in humans do not display the expected phenotype [2], [3], [4].

Interspecies divergence of gene expression is a primary cause of functional differences, but several studies have demonstrated the strong conservation of gene expression patterns among human and mouse tissues, including kidneys [5], [6], [7]. However, kidneys are heterogenous organs, the function of which results from the spatial integration of the tasks of all the successive segments constituting each nephron. Previous studies in both humans and mice have demonstrated the discrete aspect of gene expression along the nephron [8], [9], i.e., a large number of genes is specifically expressed in a single nephron segment or in the sub-segments constituting an anatomical structure. In order to determine whether divergence in gene expression along the nephron might underlie physiological differences between humans and mice, we performed a large scale comparison of gene expression profiles across the main functional structures constituting the nephron in these two species.

## Results

### Mouse and Human Kidney Transcriptome Database

We analyzed SAGE libraries previously generated in our laboratory from glomeruli (Glom), initial and terminal portions of the proximal tubule (S1 and S3), medullary and cortical thick ascending limbs of Henle’s loop (mTAL and cTAL), distal convoluted tubules (DCT) and cortical and outer medullary collecting ducts (CCD and OMCD) dissected from human [8] and mouse kidneys [9]. To our knowledge, these are the only available transcriptome data on kidney sub-structures. Because the relative abundance of a transcript-specific tag in a SAGE library reflects the abundance of the cognate transcript in the biological sample, SAGE allows for intra- and interspecies comparison of absolute gene expression levels, which is a major advantage over microarray technology for evaluating the conservation of gene expression profiles. *Per contra*, when coupled with microdissection of kidney sub-structures, SAGE remains a tedious and time-consuming technology that precludes multiple testing and assessment of assay variability. In our study, biological variability was minimized by pooling samples from several individuals (8 mice and 9 humans) in each library. Technical variability can be estimated from the comparison of two available SAGE libraries independently generated from mouse OMCD [9], [10]. The relative occurrences of tags in these two libraries were linearly correlated (slope: 1.019; correlation coefficient: 0.742; Figure S1), indicating the rather good reproducibility of the method (see also reference [11]).

The number of sequenced tags varied from 43,000 to 100,000 tags per library [8], [9]. To correct for these inter-library differences in the depth of analysis, tag abundances were normalized to 10,000 tags in each library. From these libraries, we constructed a human-mouse kidney SAGE (HMKS) database that includes the normalized tag abundance of 4644 pairs of tags unequivocally annotated as ortholog transcripts and counted at least once in a library (Table S1). The molecular diversity of human and mouse libraries was similar, with 4283 and 4184 transcript-specific tags detected at least once in human and mouse libraries respectively.

### Gene Expression Level in Human and Mouse Kidney Sub-structures

**Figure 1:**
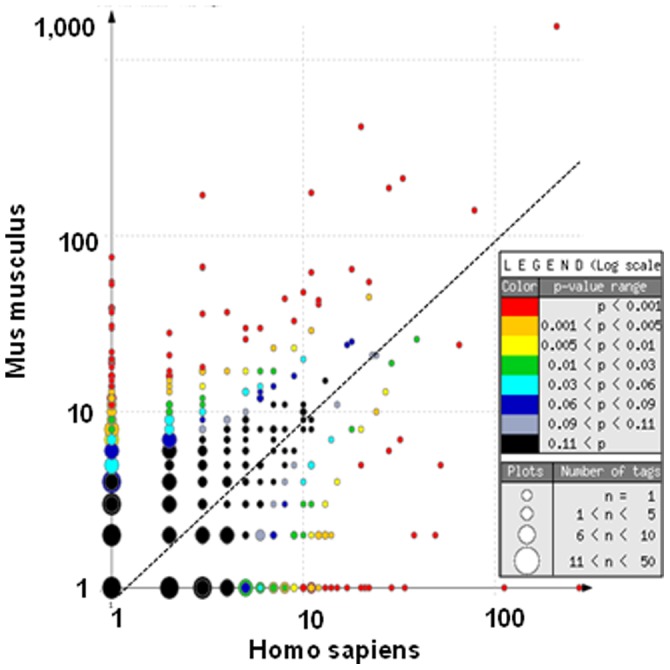
Scatter-plot of tag distribution in OMCD of mouse and human. This diagram plots the abundance of gene orthologous-specific tags in the OMCD of the two species. Data correspond to 66,374 and 70,524 tags in human and mouse kidneys respectively. The size of the spots corresponds to the number of different transcripts and their colour to the p value, as indicated in the inset. In this logarithmic scale, null abundances were plotted at a value of one.

In each kidney sub-structure, 4 to 12% of the transcripts show statistically different levels (p<0.005 by Monte Carlo test) of tag occurrence between humans and mice (Table 1). More transcripts are over-represented in the mouse kidney structures than the opposite and, accordingly, the total tag count in mouse libraries is approximately 1.5-fold that in human libraries. The scatter-plot of tag distribution in mouse and human OMCDs, displayed as an example in Figure 1, shows statistical differences in abundance for both low- and high-occurrence transcripts. Similar results were observed in all structures (Figure S2).

**Table 1:** Number of transcripts differentially expressed in human and mouse kidney structures.

	Glom	S1	S3	mTAL	cTAL	DCT	CCD	OMCD
**N**	1714 (100)	1637 (100)	1698 (100)	1848 (100)	1938 (100)	1868 (100)	1967 (100)	2090 (100)
**Hs>Mm**	25 (1.5)	69 (4.2)	50 (2.9)	29 (1.6)	44 (2.4)	55 (2.9)	32 (1.6)	53 (2.5)
**Hs<Mm**	72 (4.2)	129 (7.9)	52 (3.1)	44 (2.4)	56 (2.9)	72 (3.9)	92 (4.7)	101 (4.8)

This table lists the total number of transcripts analyzed in the different kidney structures (N) and the number of those present at statistically higher or lower levels (p<0.005) in human and mouse structures (Hs>Mm and Hs>Mm respectively). Values in parenthesis are percentages.

**Figure 2:**
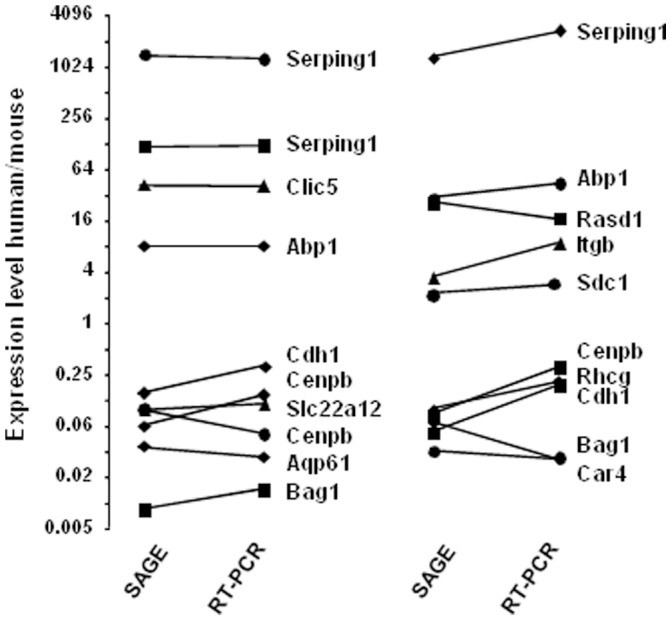
Comparison of gene expression in human and mouse kidney as determined by SAGE and RT-PCR. Expression of selected genes was determined in the glomerulus (triangles), the S1 segment (circles), the cortical thick ascending limb of Henle’s loop (squares) or the cortical collecting duct (lozenges) by either SAGE or RT-PCR. In both cases, data were normalized to Rplp1 expression. Results were calculated as the human-to-mouse ratio of expression levels (for calculations, a tag occurrence of 0.01 was taken when the tag was not detected). Data are presented according to a base 2 log scale; they were arbitrarily distributed in two panels for legibility purposes.

These interspecies differences in gene expression levels are higher than anticipated from methodological errors: Comparison of mouse and human OMCD tag abundance yields a regression line with a slope of 0.516 and a correlation coefficient of 0.147, a much lesser correlation than when comparing two mouse OMCD libraries (slope, 1.019; R^2^, 0.742; Figure S1). To further confirm interspecies SAGE-derived differences in expression levels, we compared by RT-PCR the expression levels of ∼20 randomly chosen transcripts in different mouse and human kidney sub-structures. Similar ranges of interspecies differences in transcript abundance were observed for SAGE and RT-PCR data (Figure 2).

Functional annotation of genes expressed at similar or different levels in humans and mice (Table 2) did not reveal any general pattern of gene deregulation except for: 1) the over-representation of genes involved in cell proliferation, death, biogenesis, development and protein metabolism among the genes over-expressed in human glomeruli compared with mouse glomeruli, and 2) the over-representation of genes involved in transport functions among the genes over-expressed in most nephron segments of the mouse.

**Table 2:** Functional annotation of genes expressed at different or similar levels in human and mouse kidney structures.

BiologicalProcessBins		cell adhesion	cell-cell signaling	cell cycleandproliferation	death	cell organization and biogenesis	proteinmetabolism	DNAmetabolism	RNAmetabolism	other metabolic processes	stress response	transport	developmental processes	signal transduction	N
**Glom**	Hs>Mm	0.077	0	*0.308*	*0.231*	*0.385*	*0.462*	0	0.192	0.154	0.154	0.154	*0.385*	0.308	**25**
	Hs<Mm	*0.113*	0.085	0.042	0.099	0.155	0.225	0	0.127	0.239	0.141	0.268	0.225	*0.282*	**71**
	Hs≈Mm	0.044	0.035	0.113	0.086	0.183	0.194	0.029	0.176	0.215	0.085	0.188	0.197	0.182	**1536**
**S1**	Hs>Mm	0.049	0.033	0.098	0.098	0.18	0.246	0.033	0.148	0.246	0.131	0.279	0.131	0.164	**61**
	Hs<Mm	0.042	0.051	0.119	0.093	0.178	0.203	0.034	0.110	0.331	0.119	*0.280*	0.127	0.144	**118**
	Hs≈Mm	0.035	0.028	0.096	0.069	0.176	0.189	0.024	0.144	0.210	0.078	0.195	0.165	0.150	**1458**
**S3**	Hs>Mm	0	0.040	0.100	0.140	0.240	0.280	0.040	0.140	0.280	*0.200*	0.240	0.140	0.180	**50**
	Hs<Mm	0	0.039	0.137	0.059	0.235	0.255	0	*0*	*0.490*	0.098	0.235	0.157	0.157	**51**
	Hs≈Mm	0.037	0.030	0.102	0.074	0.179	0.195	0.028	0.162	0.215	0.079	0.194	0.182	0.160	**1522**
**mTAL**	Hs>Mm	0.103	0.069	0.103	0.172	0.31	0.276	0.069	0.310	0.207	0.172	0.276	0.276	0.276	**29**
	Hs<Mm	0.049	0.098	0.171	0.122	0.293	0.195	0	0.171	*0.341*	0.171	*0.366*	0.293	0.293	**41**
	Hs≈Mm	0.037	0.031	0.112	0.077	0.180	0.203	0.022	0.178	0.202	0.083	0.200	0.194	0.166	**1642**
**cTAL**	Hs>Mm	0.100	*0.100*	0.125	*0.175*	0.275	0.300	0.050	*0.325*	0.250	*0.175*	0.250	0.325	0.150	**40**
	Hs<Mm	0.038	*0.096*	0.154	0.115	0.250	0.250	0	0.154	*0.365*	*0.192*	*0.365*	0.173	*0.288*	**52**
	Hs≈Mm	0.037	0.028	0.108	0.074	0.180	0.193	0.027	0.180	0.198	0.076	0.194	0.190	0.165	**1723**
**DCT**	Hs>Mm	0.057	0.038	0.038	0.075	0.151	0.264	0	0.208	0.264	0.094	0.245	0.189	0.132	**53**
	Hs<Mm	*0.092*	*0.108*	0.169	0.092	0.246	0.262	0	0.154	0.308	0.108	*0.400*	*0.292*	*0.292*	**65**
	Hs≈Mm	0.036	0.028	0.109	0.073	0.168	0.190	0.029	0.169	0.209	0.078	0.194	0.180	0.165	**1668**
**CCD**	Hs>Mm	0.067	0	0.167	0.133	0.200	0.267	0	0.300	0.233	0.167	0.133	0.233	0.167	**30**
	Hs<Mm	0.047	0.059	0.082	0.118	0.224	0.188	0	0.129	0.235	0.153	*0.376*	0.176	0.235	**85**
	Hs≈Mm	0.035	0.028	0.106	0.077	0.174	0.186	0.027	0.174	0.213	0.079	0.185	0.184	0.166	**1749**
**OMCD**	Hs>Mm	0.059	0.039	0.098	*0.176*	0.255	0.176	0	0.196	0.314	0.137	0.157	0.196	0.255	**51**
	Hs<Mm	0.071	*0.081*	0.172	*0.172*	0.253	0.172	0.020	0.101	0.263	*0.152*	*0.343*	0.232	*0.263*	**99**
	Hs≈Mm	0.039	0.031	0.111	0.081	0.177	0.201	0.028	0.175	0.201	0.077	0.185	0.188	0.171	**1859**

Genes expressed at statistically (p<0.005) higher (Hs>Mm) or lower (Hs<Mm) levels in humans, relative to mice, or at not statistically different levels (p>0.005) (Hs≈Mm) were assigned to biological processes using MGI GO_Slim Chart Tool. The number of genes in each process group is expressed as a fraction of the total number of genes in each class (N). Figures in italics indicate values statistically different from the Hs≈Mm group (chi square test, p<0.05). Note that N values in this table are slightly different from those shown in table 1 because genes that were not recognized by GO_Slim Chart Tool were dismissed.

### Gene Expression Profile along the Human and Mouse Nephron

The specificity of renal function depends not only on the level of expression of genes but also on their specific patterns of expression along the nephron. Gene expression profiles may be compared using two classical mathematical tools: Pearson’s correlation coefficient r (or Pearson’s distance, calculated as 1– r) [12] and the Euclidean distance d [13], and we used these two tools.

**Figure 3:**
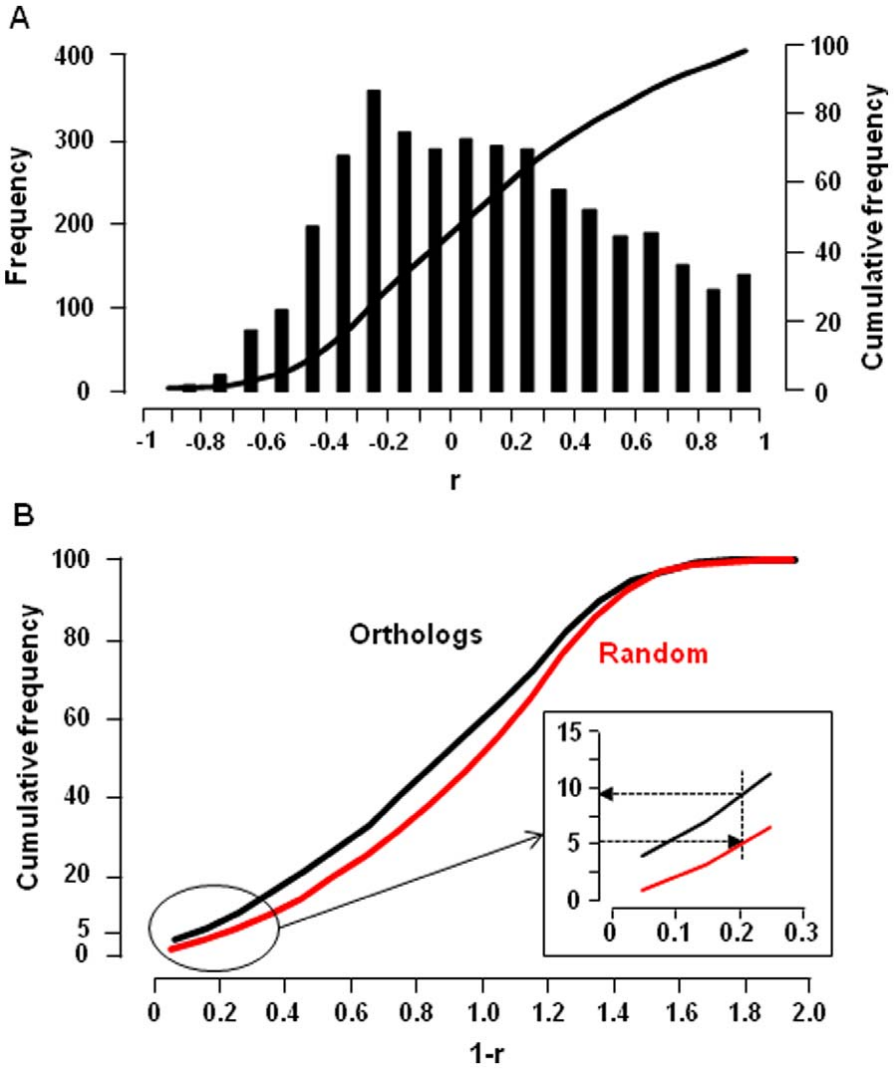
Comparison of gene expression profiles in human and mouse nephron. **A.** The columns show the histogram of distribution of the Pearson’s correlation coefficients (r) of the 4644 genes in the HMKS database. The curve shows the cumulative fraction. **B.** The curves show the cumulative distribution of Pearson’s distances (1-r) for pairs of orthologous genes and random-paired genes. The inset shows that 9.5% of orthologous transcripts displayed a Pearson’s distance smaller than that of the 5% random pairs of transcripts with the lowest Pearson’s distances.

For each pair of genes, Pearson’s correlation coefficient r was calculated as:
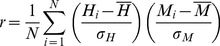
(1)where H_i_ and M_i_ are the tag abundance in sub-structure i of human and mouse kidney respectively, 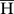 and 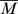 are the mean tag abundance in the N human and mouse libraries respectively, and σ_H_ and σ_M_ are the standard deviation of these means. Pearson’s coefficient can be calculated only for transcripts detected at least in one structure in both species. It varies from −1 to +1 so that Pearson’s distance varies from 0 (identical expression profile) to 2 (opposite distribution profile). Among the 4644 pairs of transcript orthologs in the HMKS database, 3823 had a computable r, among which >40% showed a negative r and <10% had a r>0.7 (Figure 3A). This signifies a rather low conservation of expression patterns between humans and mice orthologs in kidney sub-structures. An interesting approach for quantifying this conservation is to compare the expression profiles of pairs of orthologous genes and of random-paired genes [14]. Although statistically different (p<0.001, Mann-Whitney U test), distributions of Pearson’s distances for random-paired human and mouse transcripts and for orthologs were only moderately shifted (Figure 3B): the mean Pearson’s distance was 0.887 and 0.989 and the median distance was 0.923 and 1.042 for orthologous and random-paired human and mouse transcripts respectively. Figure 3B shows that only 9.5% of orthologous transcripts displayed a Pearson’s distance smaller than that of the 5% random pairs of transcripts with the lowest Pearson’s distances. This indicates that less than 10% of orthologous transcripts show a higher conservation of expression profiles than the neutral expectation at the p<0.05 significance level.

Pearson’s correlation coefficient tends to show low conservation of expression profiles for genes with relatively uniform expression profiles across tissues [15]. The use of the Euclidean distance circumvents in part this bias. For each pair of genes, the Euclidean distance was calculated as
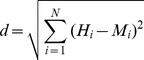
(2)


Euclidean distances vary from 0, for genes with identical expression profiles, to infinity for genes with poor conservation of expression profiles and/or large differences in absolute expression levels across species. This range of variation can be reduced through normalization of gene expression levels prior to calculation of Euclidean distances. As previously described [14], tag abundances were normalized within each species using the relative expression (RE) computed as:
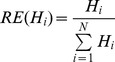
(3a)

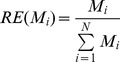
(3b)


**Figure 4:**
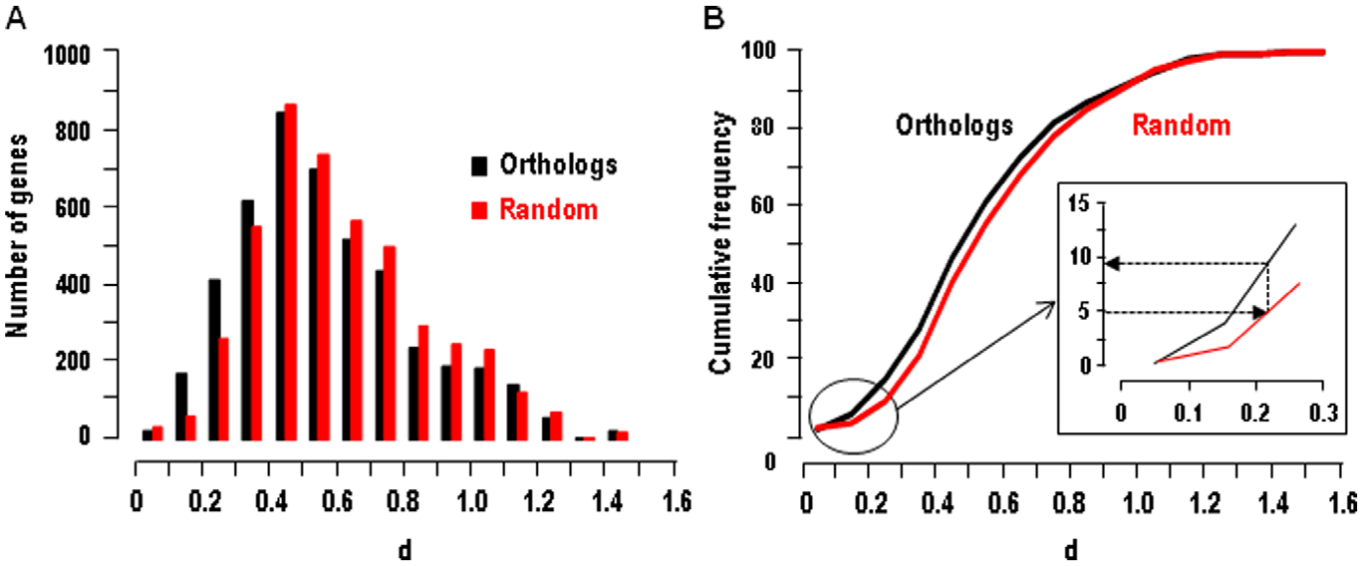
Comparison of gene expression profiles in human and mouse nephron. **A.** The columns show the histogram of distribution of the Euclidean distances d of the 4644 pairs of orthologs genes in the HMKS database and of random associated pairs of the same genes. **B.** Cumulative distribution of Euclidean distances d for pairs of orthologous genes and random-paired genes. The inset shows that 9.3% of orthologous transcripts displayed an Euclidean distance smaller than that of the 5% random pairs of transcripts with the lowest Euclidean distances.

This normalization procedure sets to one the sum of expression levels of each gene in the different kidney sub-structures in each species, and therefore abolishes the intra- and interspecies differences in absolute expression levels. As a consequence, Euclidean distances calculated from normalized data vary from 0 to 2. Figure 4A shows that, although statistically different (p<0.001, Mann-Whitney U test), the distribution pattern of Euclidean distances for orthologous transcripts was only slightly shifted relative to that of random-paired transcripts (mean: 0.574 vs. 0.610; median: 0.527 vs 0.563 for ortholog and random-paired transcripts respectively). Accordingly, only 9.3% of orthologous transcripts showed a higher conservation of expression profiles than the neutral expectation at the p<0.05 significance level (Figure 4B).

### Hierarchical Clustering

**Figure 5:**
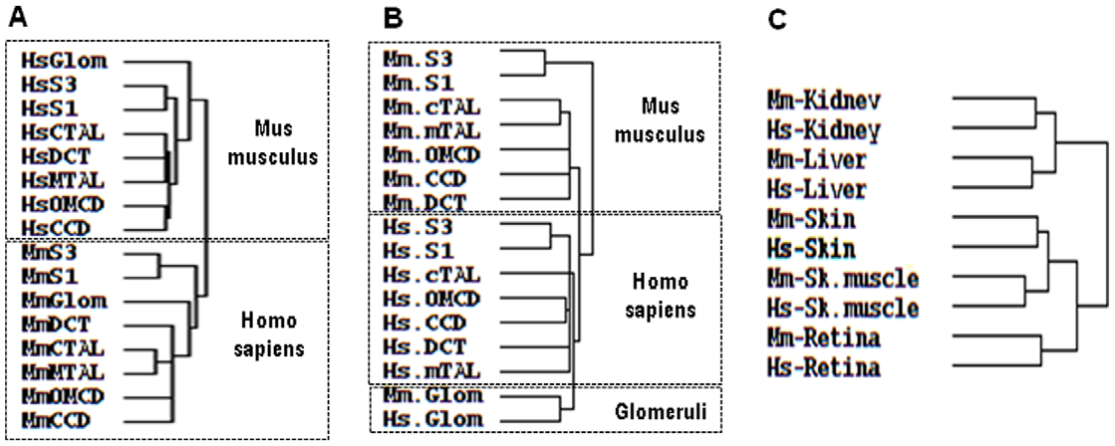
Hierarchical clustering of gene expression in human and mouse kidney sub-structures and tissues. Clustering was performed using Cluster. The dendograms showing relationships between libraries were graphically visualized using TreeView. **A.** Clustering according to tag abundance of the 4644 ortholog gene-specific tags present in the HMKS database discloses that the two main subgroups of structures with similar patterns of gene expression correspond to human and mouse structures respectively. **B.** Clustering according to relative abundance of the same tags reveals similarities between human and mouse glomuruli but not other sub-structures. **C.** Clustering according to relative abundance of the 5145 pairs of orthologous genes detected in different SAGE libraries from human and mouse tissues (see Table S2) reveals interspecies conservation of expression profiles.

Hierarchical clustering of the 4644 transcripts from HMKS database confirmed this finding as it unexpectedly revealed a greater similarity between different kidney sub-structures within a given species than between cognate structures in the two species (Figure 5A). Thus, a human glomerulus resembles more a human proximal tubule than a mouse glomerulus in terms of gene expression patterns. Conversely, it confirmed the similarities between the sub-segments constituting either the proximal tubule (S1 and S3), the thick ascending limb of Henle’s loop (mTAL and cTAL) or the collecting duct (CCD and OMCD) within a given species [8], [9]. Normalization of expression levels (relative expression), which suppresses intra- and interspecies differences in gene expression levels (see above), revealed a similarity between human and mouse glomerulus, but not between other human and mouse sub-structures (Figure 5B). This indicates that part of the divergence in gene expression profiles observed between human and mouse kidney sub-structures stems from differences in absolute levels of expression.

To demonstrate that this lack of conservation of gene expression profiles between human and mouse kidney sub-structures is not of methodological origin, such as the SAGE source of the data, the data treatment, or the clustering procedure, we used the same approaches to analyze SAGE libraries generated from human and mouse kidney, retina, skeletal muscle, liver and skin (Table S2). Among the 5145 pairs of ortholog transcripts detected in at least one of these 5 libraries, 4131 had computable Pearson’s coefficients, among which 23% were >0.7 (versus <10% in kidney sub-structures). Moreover, hierarchical clustering revealed a higher conservation of gene expression profiles across cognate tissues from the two species than across tissues of each species (Figure 5C).

**Figure 6:**
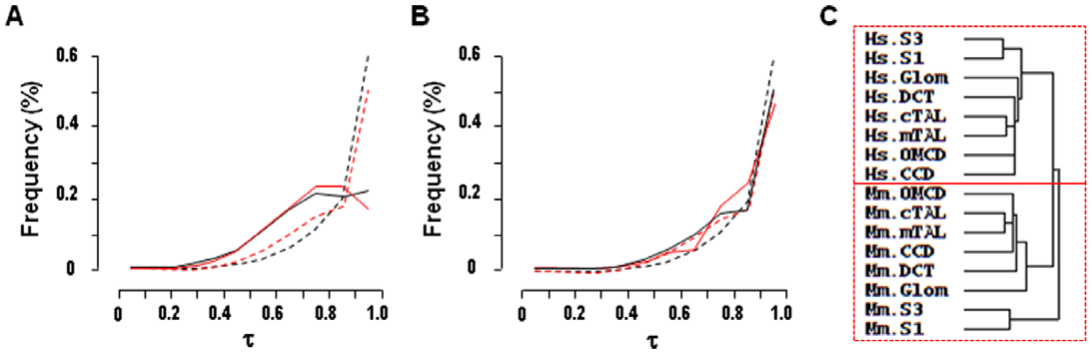
Specificity of expression of genes in human and mouse kidney sub-structures and tissues. A. Distribution of expression specificity (τ) of genes present in HMKS database (full lines) and in SAGE libraries from tissues (Table S2, stippled lines). The black and red lines correspond to human and mouse genes respectively. **B.** Distribution of τ for 1943 genes from HMKS (full lines) selected to display a similar profile of τ distribution as genes in the SAGE libraries from tissues (stippled lines). **C.** Hierarchical clustering of the 1943 selected genes from HMKS with high expression specificity confirms the divergence in gene expression profile between human and mouse kidney sub-structures.

It was reported recently that the presence of uniformly expressed genes may bias the analysis of conservation of gene expression [16]. To address this issue, we computed the expression specificity of genes (τ) in each species as previously described [17]:
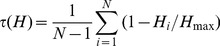
(4a)

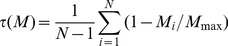
(4b)where H_i_ and M_i_ are the tag abundance in kidney sub-structure or tissue i of human and mouse respectively, and H_max_ and M_max_ are the maximal tag abundance in the N human and mouse libraries respectively. τ varies from 0, for uniformly expressed genes, to 1 for genes specifically expressed in a single kidney sub-structure or tissue. Results in figure 6A indicate that 64% of orthologs from both human and mouse kidney sub-structures displayed a τ>0.7, indicating a rather high degree of expression specificity. However, genes from human and mouse tissues analyzed in figure 5C displayed a higher specificity of expression as over 80% among them had a τ>0.7. To evaluate the impact of expression specificity, we selected a subgroup of 1943 orthologs from kidney sub-structures which displayed the same profile of expression specificity as the genes from human and mouse tissues analyzed in figure 5C (Figure 6B). Functional clustering of these 1943 orthologs confirmed the lack of conservation of gene expression profiles between human and mouse kidney sub-structures (Figure 6C).

**Figure 7:**
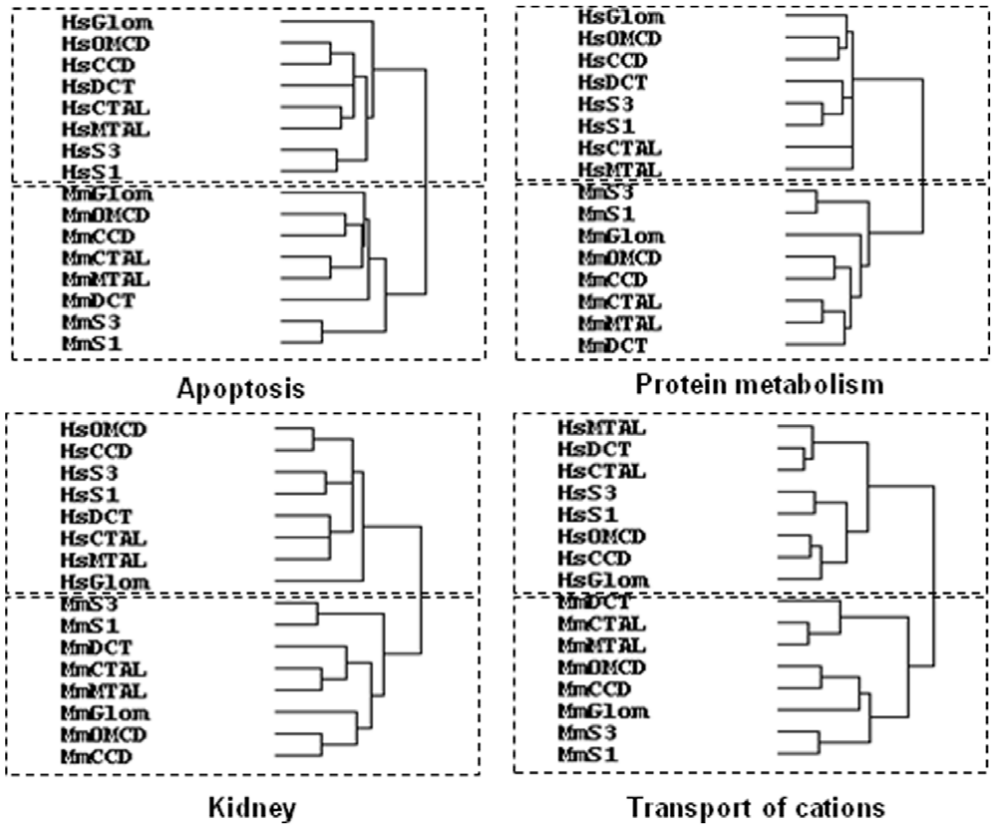
Hierarchical clustering of functionally-related groups of genes expressed in human and mouse kidney structures. Functionally related groups of genes were defined according to the ontology of Ingenuity Pathway Analysis. The four groups selected included 2305 (apoptosis), 780 (protein metabolism), 1525 (kidney) and 402 genes (cation transport). The HMSK database contained 706, 305, 494 and 100 genes corresponding to these different functional groups respectively (see list of genes in Table S2) which were clustered as in figure 4. Here again, the two main clusters are defined by the species rather than the kidney structure.

The apparent dissimilarity in gene expression patterns along human and mouse kidney sub-structures may stem also from the fact that a large fraction of the 4644 genes present in the HMKS database are involved in housekeeping rather than kidney specific functions, and that housekeeping genes show less interspecies tissue specificity conservation [6]. To evaluate this hypothesis, we performed the hierarchical clustering of groups of transcripts related to either kidney-specific or housekeeping functions. The functional groups were selected from Ingenuity Pathway Analysis (IPA) ontology. For kidney-specific functions we selected the “kidney” and “transport of cations” clusters which respectively contain 1525 and 402 genes, among which 494 and 100 were present in the HMKS database (Table S3). For housekeeping functions we selected the “apoptosis” and “protein metabolism” clusters containing respectively 2305 and 780 genes, 706 and 305 of which were present in the HMKS database (Table S3). Hierarchical clustering of these four groups of genes yielded similar results, with a higher degree of similarity between different structures within a same species than between cognate structures in the two species (Figure 7). Interestingly, within a given species, this analysis revealed similar degrees of similarities between functionally related structures for kidney-specific and housekeeping genes. Similar results were obtained for other functional groups of genes: transport of anions (n = 22), hormones (n = 83), epithelium (n = 96) or differentiation (n = 424) (data not shown).

**Figure 8:**
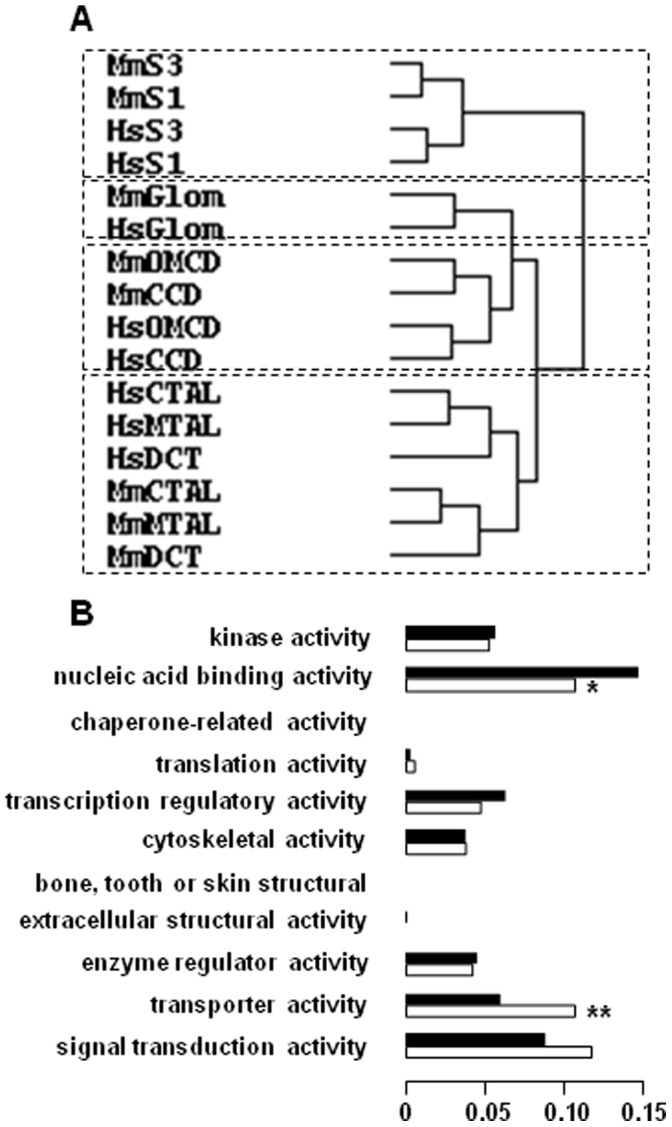
Hierarchical and functional clustering of genes with similar expression patterns along human and mouse nephrons. Genes with similar patterns of expression in human and mouse nephrons (i.e., with a Pearson’s coefficient >0.7) were selected within the HMKS database. **A.** Hierarchical clustering of the 419 selected genes was performed as in figure 4. **B.** Functional clustering of the 419 selected genes (open bars, see list in Table S2) and all the 4644 genes present in the HMKS database was performed using the GO_Slim Chart Tool at MGI. Data are expressed as fractions of the total number of genes. Values statistically different between the two sets of genes were determined by the chi square test: *, p<0.05; **, p<0.001.

Finally, we attempted to characterize a molecular signature that is conserved in mouse and human kidneys. For this purpose, we selected the 419 genes showing similar patterns of expression along the human and mouse nephron, as evaluated by a Pearson’s coefficient >0.7 (Table S3). As expected, hierarchical clustering of these genes revealed interspecies similarities between groups of cognate structures (Figure 8A). However, within a subgroup of structures (e.g. the S1 and S3 sub-segments of the proximal tubule), the similarity was higher between sub-segments of a same species than between cognate structures in the two species. Functional clustering of this kidney-specific group of genes revealed an over-representation of genes associated with transport functions when compared to all the genes in the HMKS database (Figure 8B).

## Discussion

Annotation of previously published SAGE libraries [8], [9] allowed us to identify over 4500 pairs of orthologous transcripts expressed in the mouse and/or human kidney. Analysis of this database reveals marked differences between human and mouse kidneys in terms of levels and patterns of gene expression along the nephron, resulting in a low level of conservation of gene expression between their constitutive sub-structures. Three aspects of this conclusion deserve further discussion: 1. the difference in gene expression levels between human and mouse; 2. whether the paradoxical conclusion regarding the low conservation of gene expression among human and mouse kidney sub-structures may stem from the very nature of the data or their treatment procedure; and 3. what might explain the paradox.

### Interspecies Differences in Gene Expression Level

Many reports show large interspecies differences in the levels of expression of functionally related groups of genes such as ion channels, transcription factors or glutathione metabolism [18], [19], [20]. However, most studies that addressed interspecies conservation of gene expression do not consider these quantitative differences because they are based on oligonucleotide microarray analysis of gene expression, a technology that does not allow for meaningful comparison of the absolute levels of gene expression. This stems mainly from the fact that the transcript-specific probes within a same array or among species-specific arrays display different affinities for their cognate transcripts. Quite curiously, in recent studies based on RNA sequencing, a theoretically quantitative technology (the relative occurrence of sequenced transcript-specific RNA fragments is proportional to the abundance of the corresponding transcripts in the original tissue sample), the authors did not consider absolute levels of gene expression either, as they normalized data to relative expression levels prior to their analysis [5]. SAGE also is theoretically a quantitative method and, accordingly, differences in gene expression levels observed between human and mouse kidney sub-structures were confirmed by RT-qPCR.

Our data show that most segments of the mouse nephron display a higher expression level of genes related to transport functions. It is generally assumed that ion transport is more intense in mouse kidneys than in human kidneys, owing to the higher metabolic rate and blood flow per body weight unit in the former species. However, data summarized in table 3 indicate that this is not the case when kidney function is expressed as a function of kidney mass, due to the higher kidney-to-body mass ratio, and greater food and water intake, and therefore excretion, in mice compared to humans. For example, as illustrated in table 3, water and sodium reabsorption rates per g of kidney mass are of the same order of magnitude in both species. Therefore, the quantitative differences between mice and humans in the expression level of transport genes might be related to other functional differences, such as a slower mRNA/protein turnover in humans compared with mice.

**Table 3:** Comparison of structural and functional features in human and mouse kidneys.

	Human	Mouse
Body weight	60 kg	25 g
Kidney weight	320 g	320 mg
	5.3 g/kg BW	12 g/kg BW
Number of nephrons	2×10^6^	10^4^
	33/g BW	400/g BW
	6/mg KW	31/mg KW
GFR	125 ml/min	120 µl/min
	2.1 ml/min/kg BW	4.8 ml/min/kg BW
	391 µl/min/g KW	375 µl/min/g KW
Water intake and excretion	1.5 l/day	5 ml/day
Water reabsorption	178.5 l/day	168 ml/day
	0.56 l/day/g KW	0.52 l/day/g KW
Sodium intake and excretion	5 g/day	15 mg/day
Filtered sodium	580 g/day	556 mg/day
Sodium reabsorption	575 g/day	541 mg/day
	1.8 g/day/g KW	1.7 g/day/g KW

Data for body and kidney weights (BW & KW), glomerular filtration rate (GFR), number of nephrons (for both kidneys), urine volume (or water excretion) and sodium intake are commonly accepted values for humans. For mice, KW and GFR are from [10]; the GFR is calculated by multiplying the single nephron GFR of 10 nl/min [27] by the number of nephrons; water and sodium intake are unpublished data from our laboratory.

We recently reported that in the mouse kidney, glomerulus epithelial cells undergo proliferation under basal conditions, as opposed to tubular epithelial cells which are quiescent [9]. The higher expression level of genes related to proliferation/differentiation processes observed in human versus mouse glomeruli suggests that proliferation processes are more active in human than mouse glomeruli. The proliferative capacity of cells from the parietal sheet of Bowman’s capsule is enhanced during glomerular pathologies such as focal segmental glomerulosclerosis [21]. The difference between humans and mice may therefore stem from the glomerular health status of the two populations, particularly because the patients analyzed in this study were much older (59±10 years), in relative terms, than the mice (8–10 weeks), and therefore more prone to display glomerular pathologies. Because human samples emanated from healthy kidney fragments of donors who had undergone surgery for removal of kidney tumors [8], one might argue also that the presence of the tumor, even at distance from the analyzed sample, may have triggered the expression of proliferative genes. However, this seems unlikely because such a deregulation of gene expression would be expected to affect all nephron structures and not specifically the glomerulus.

### Impact of Data Treatment on the Conclusion regarding Interspecies Divergence of Gene Expression Profiles

Comparison of gene expression profiles is classically assessed either by Pearson’s distance or by the Euclidean distance [12], [13], and some studies demonstrated that the former measurements tends to increase the divergence for genes with relatively uniform patterns of expression as compared to the latter [15]. In the present study, we reached the same conclusion when using either one of the two measures, as 9.5% and 9.3% of orthologous transcripts show a higher conservation of expression profiles than the neutral expectation (at p<0.05) when using Pearson’s and Euclidean distances respectively. More recently, it was reported that both Pearson’s and Euclidean distances may be unsuited to measure the expression distance of genes with low expression specificity [16]. However, we found that excluding genes with low expression specificity from the HMKS database changed neither the hierarchical clustering nor our conclusion regarding the low conservation of expression profile between human and mouse kidney sub-structures.

The impact of data treatment is evidenced by the work of Liao and Zhang [14] who re-analyzed a set of microarray data from human and mouse tissues previously analyzed by Yanai et al. [22], and reached the opposite conclusion, namely, conservation instead of divergence of expression profiles between the two species. Liao and Zhang’s conclusion is based on the comparison of Euclidean distances after two operations: the calculation of relative abundance, a prerequisite when analyzing microarray data (see above), and the correction for measurement errors in expression profiles. The latter is justified by their finding that the differences in expression between different probes for the same gene in a given species are only slightly lower than differences between pairs of orthologs. Consequently they corrected the interspecies distance between pairs of orthologs by subtracting the mean value of intraspecies distances (the errors). This correction is debatable as 1. It gives rise to a large proportion of orthologs displaying “negative” corrected distances (when the measurement error is higher than the detected interspecies difference), the meaning of which remains unclear, and 2. Because the error is subtracted, the higher the measurement error is, the seemingly higher the conservation in gene expression. Their final conclusion is further biased because it is based on the comparison between the corrected distances for pairs of orthologs (which are artificially low) and the non corrected distances for random-paired genes. What their study implies, in fact, is that no conclusion regarding conservation of expression patterns between human and mouse tissues can be derived from their dataset, because the uncertainty on measurements of expression level is too high.

Nonetheless, Liao and Zhang’s study points out the importance of considering the errors in the determination of gene expression profiles, which may be high when using large scale methodologies. For economic/technical reasons, we used in this study a single set of data for each tissue and could not evaluate quantification errors. Comparison of two independent mouse OMCD libraries and a human OMCD library revealed much higher intraspecies, relative to interspecies, reproducibility, but we could not conclude on the reproducibility of gene profiling. Nonetheless, we ascertained the global validity of our data treatment method by confirming, with this same method, the interspecies conservation of gene expression patterns among different tissues previously demonstrated using different experimental and data analysis approaches [5], [6], [7].

### Differences in Conservation of Gene Expression Patterns within the Kidney and Among Different Tissues

There is an apparent paradox between the high conservation of gene expression in kidney compared to other tissues and the large divergence among kidney sub-structures. The main function of nephrons is to transport solutes and water to maintain homeostasis. Large differences in expression profiles of solute carriers (SLC family), organic anion transporters (SLCO family) and ATP binding cassette transporters (ABC family) were described across human, mouse, dog and monkey tissues [23]. The same was observed within the kidney since clustering of genes related to cation or anion transport revealed more intra- than interspecies similarity among kidney sub-structures. There are however obvious exceptions to this rule since the conserved genomic signature of the kidney includes a large fraction of transport-related genes which display some of the highest correlation coefficients, e.g. the furosemide- and thiazide-sensitive NaCl transporters (SlC12A1 and SLC12A3 respectively, Pearson’s coefficient >0.99), water channels (AQP2, 3 & 6, Pearson’s coefficient >0.97) or the cell junction proteins claudins which control the paracellular transport pathway (CLDN1, 2 & 5, Pearson’s coefficient >0.94). In addition, the genes with conserved expression patterns between human and mouse kidney sub-structures contain a proportion of transport-related genes that is above the neutral expectation at the p<0.05 significance level.

The paradox behind the differences in intra-kidney and inter-tissue conservation of gene expression may stem from the fact that, unlike other organs, all kidney sub-structures except glomeruli basically perform the same function (solutes and water transport). In other words, there should be more conservation of gene expression between kidney sub-structures than between different organs within a given species. Consequently, intra-species conservation is stronger for kidney sub-structures than for functionally unrelated tissues, and conversely inter-species conservation is weaker for kidney sub-structures than for other tissues. This is illustrated by our finding that when the impact of absolute expression level is abolished through data normalization, the only sub-structures that co-clusterize among human and mouse are glomeruli, the structure that most deeply differs structurally and functionally from others.

In summary, this study shows that although there is a global conservation in gene expression between human and mouse kidney at the whole organ scale, there are marked differences in both the levels and the patterns of expression at the scale of the different sub-structures constituting the nephron. Conservation and divergence of gene expression at these two scales likely account for the fact that, although kidneys assume the same global function in the two species, genetic or toxicologic mouse “models” of human pathologies do not display the expected phenotype. This study emphasizes that much caution should be taken when extrapolating results from mouse kidney to human kidney and *vice versa*.

## Methods

### Construction of SAGE Libraries Database

A database was built with the public (Cancer Genome Anatomy Project, NIH) and SAGE libraries (Gene Expression Omnibus accession nos GSE25223, GSM10419 and GSM10423–10429). SAGE libraries are relative to the human and mouse kidney and share the same anchor enzyme, Sau3A1. Because of differences in SAGE protocols (10 bp- and 17 bp-SAGE-tags) and to facilitate *in silico* comparison, the length of tags in the mouse library was reduced to 10 bp.

### Annotation of SAGE Libraries

Human and mouse tag annotation libraries were constructed from reference genes from the UniGene data (NCBI: UniGene Homo sapiens built n°206 & UniGene Mus musculus built n°166). For each sequence of these databases, the canonical SAGE tag located downstream the 3′-most Sau3AI restriction site (GATC) of the sequence (R1) as well as putative tags located in inner positions (labeled as R2, R3 and R4 starting from the 3′ end of the transcript) were extracted. From each anchoring site, virtual SAGE tags from the complementary strand, hereafter called “antisense tags” (AS1–AS4), were extracted also. From there, experimental tags obtained from SAGE libraries were matched and annotated using their respective database. Experimental tags matching several virtual tags were annotated either as multiple matches if the different annotations corresponded to unrelated transcripts or as a single transcript when the different annotations corresponded to variants of that transcript.

### Interspecies Comparison of SAGE Libraries

HomoloGene database (http://www.ncbi.nlm.nih.gov/homologene, built n°56) is a system for automated detection of homologs among the annotated genes of several completely sequenced eukaryotic genomes. Human and mouse homologene groups contain respectively 18,876 and 19,026 genes. Therefore, only the annotated genes in Mouse and Human with a well-described homologous gene could be cross-checked. An internal algorithm allows the comparison between different libraries and measures the significance threshold for the observed variations [24].

### Data Clustering

Hierarchical clustering was performed using the Cluster software [25], a hierarchical average linkage clustering algorithm freely available on the web (http://rana.lbl.gov/EisenSoftware.htm). This algorithm used an iterated, agglomerative process of similarity measurements based on the Pearson correlation. In each iterative step of the algorithm, the two most similar data elements (i.e. expression profiles) were joined by a dendrogram node, after which the joined elements were averaged and replaced by a pseudo-element which was used in all subsequent iterations [25]. Results from Cluster data treatment were graphically visualized using the TreeView software also freely available at the same web address.

Functional clustering according to “process” or “function” ontology was performed using the GO_Slim Chart Tool available as a Mouse Genome Informatics (MGI) resource (http://www.informatics.jax.org/) [26]. Clusters of functionally related genes were taken from IPA (Ingenuity Systems, www.ingenuity.com).

### Microdissection

Glomeruli, initial portion of proximal tubules (S1), cortical thick ascending limbs of Henle’s loop (cTAL) and cortical collecting ducts (CCD) were microdissected from liberase-treated mouse kidneys, as previously reported [9]. Briefly, the left kidney was perfused *in situ* with 6 ml of Hank’s solution supplemented with 1 mM glutamine, 1 mM pyruvate, 0.5 mM MgCl_2_, 0.1% bovine serum albumin, 20 mM Hepes, and 0.015% liberase (Blendzyme 2, Roche Diagnostics, Meylan, France), pH 7.4. Thin pyramids were cut from the kidney and incubated in 0.006% liberase solution for 20–25 min at 30°C, and thoroughly rinsed in microdissection solution. All medium were prepared and used in an RNase-free environment. Experimental protocol was approved by our local ethic committe at Cordelier’s Research Center and performed under the supervision of an authorized experimenter (AD, license # 75-699 renewal). The cognate structures from human kidneys had been dissected previously using a similar procedure except that collagenase was substituted for liberase [8]. They were obtained from the same kidneys that were used for building SAGE libraries [8] and had been stored in liquid nitrogen until RNA extraction.

### mRNA Extraction and RT-real Time PCR Analysis

RNAs were extracted, according to the technique previously described [9], from pools of 20–50 glomeruli or nephron segments. RNAs were reverse transcribed using the first strand cDNA synthesis kit for RT-PCR (Roche Diagnostics, Meylan, France), according to the manufacturers’ protocol. Real time PCR was performed on a LightCycler (Roche Diagnostics) with the LightCycler FastStart DNA Master SYBR Green 1 kit (Roche Diagnostics) according to the manufacturers’ protocols, except that the reaction volume was reduced 2.5-fold. Specific primers (Table S4) were designed using ProbeDesign 2 (Roche Diagnostics). No DNA was detectable in samples that did not undergo reverse transcription, and in blanks run without cDNA. In each experiment, a standardization curve was made using serial dilutions of standard cDNA stock solutions made from mouse or human whole kidney RNAs, and the amount of PCR product was calculated as percent of the standard DNA. In order to compare the expression level of orthologous genes in human and mouse samples, data were standardized using RPLP1, a transcript showing similar expression levels in all structures from both mouse and human kidneys (see Table S1).

## Supporting Information

Figure S1 **Correlation between tag abundance in two mouse OMCD libraries and a human and a mouse OMCD library**. Tag abundance (normalized to 10,000 tags in each library) in a mouse OMCD SAGE library (Mm.1) was plotted against tag abundance in another mouse OMCD library (Mm.2, red dots) or a human OMCD library (Hs, black dots). The three libraries were generated independently.
(TIF)

Figure S2 **Scatter-plot of tag distribution in kidney structures of mouse and human.** This diagram plots the abundance of gene orthologous-specific tags in the glomerulus (Glom, initial and late parts of the proximal tubule (S1 and S3), medullary and cortical thick ascending limb of Henle’s loop (mTAL and cTAL), dictal convoluted tubule (DCT) and cortical and outer medullary collecting duct (CCD and OMCD) of the two species. The size of the spots corresponds to the number of different transcripts and their color to the p value, as indicated in the inset. In this logarithmic scale, null abundances were plotted at a value of one.
(TIF)

Table S1 **mRNA SAGE tags detected in the human and mouse nephron**. Values are normalized to10,000 tags in each library. gb, Genbank reference of the sequence. Genes are identified according to the HUGO nomenclature, including the symbol and the sequence definition.
(XLS)

Table S2 **mRNA SAGE tags detected in the human and mouse retina (R), skeletal muscle (SM), liver (L), skin (S) and kidney (K).** Data are from Gene Expression Omnibus accession nos GSM384124, GSM383920, GSM383907, GSM384136, GSM383901, GSM384367, GSM137114, GSM137106, GSM137111 and GSM106656. Values are normalized to 10,000 tags in each library. Genes are identified according to the HUGO symbol. Pearson’s coefficients were calculated as indicated in the results section.
(XLS)

Table S3 **Subgroups of genes from HMKS database.** Genes were selected from HMKS database on basis of their belonging to IPA functional clusters (Apoptosis, protein metabolism, kidney and, transport of cations) or of a Pearson’s coefficient >0.7. Genes are listed according to their HUGO nomenclature.
(XLSX)

Table S4 **Sequence of primers used for PCR in human and mouse kidney samples**. Genes are listed according to their HUGO nomenclature. Primers were designed with ProbeDesign 2 on basis of the listed Genbank references (gb).
(XLS)
