# Supplementary figures and images for: Of Mice and Men: Divergence of Gene Expression Patterns in Kidney
Source: PLoS One. 2012 Oct 3;7(10):e46876. doi: 10.1371/journal.pone.0046876 (PMC3463552; doi:10.1371/journal.pone.0046876)

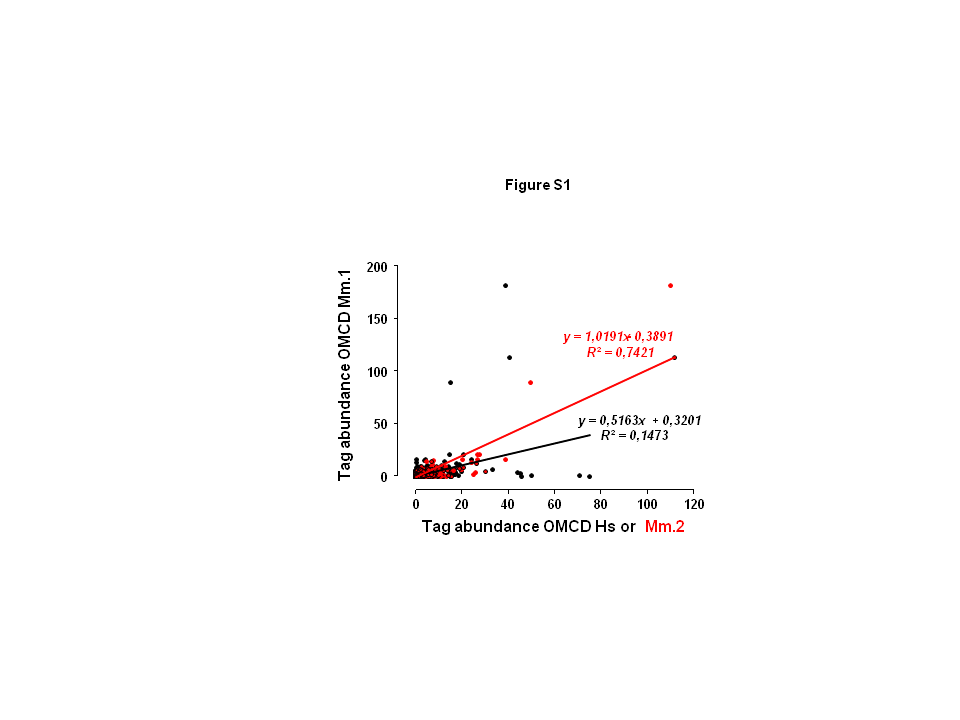

Supplement: Figure S1 — Correlation between tag abundance in two mouse OMCD libraries and a human and a mouse OMCD library. Tag abundance (normalized to 10,000 tags in each library) in a mouse OMCD SAGE library (Mm.1) was plotted against tag abundance in another mouse OMCD library (Mm.2, red dots) or a human OMCD library (Hs, black dots). The three libraries were generated independently. (TIF) [file pone.0046876.s001.tif]

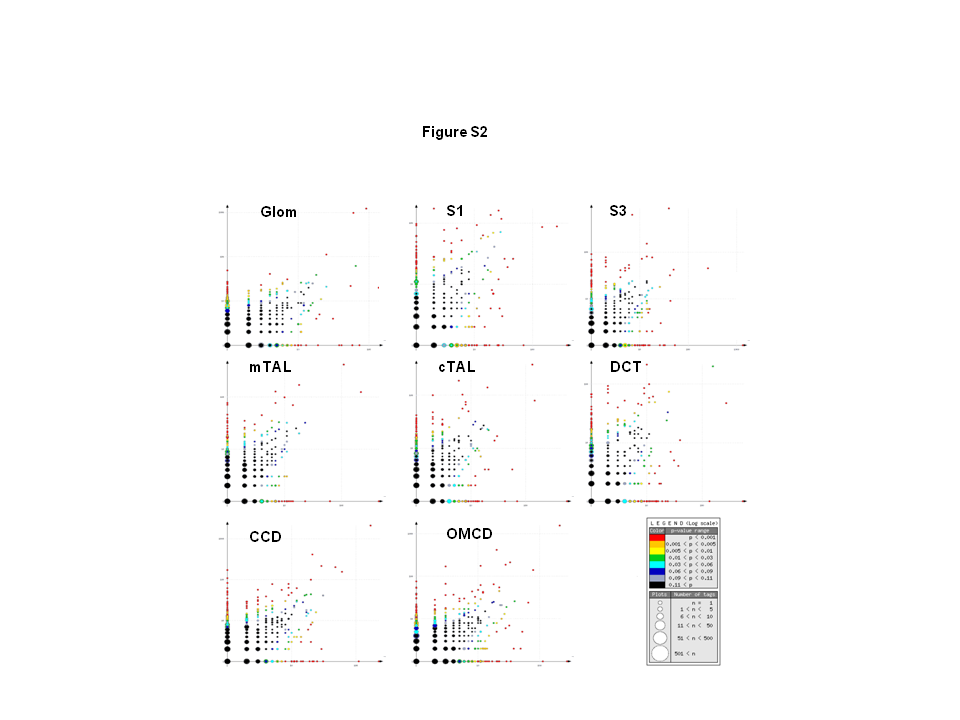

Supplement: Figure S2 — Scatter-plot of tag distribution in kidney structures of mouse and human. This diagram plots the abundance of gene orthologous-specific tags in the glomerulus (Glom, initial and late parts of the proximal tubule (S1 and S3), medullary and cortical thick ascending limb of Henle’s loop (mTAL and cTAL), dictal convoluted tubule (DCT) and cortical and outer medullary collecting duct (CCD and OMCD) of the two species. The size of the spots corresponds to the number of different transcripts and their color to the p value, as indicated in the inset. In this logarithmic scale, null abundances were plotted at a value of one. (TIF) [file pone.0046876.s002.tif]
